# Peripartum Cardiac Arrest with Terminal QRS Distortion: A Case Report

**DOI:** 10.5811/cpcem.1323

**Published:** 2023-12-06

**Authors:** Timothy D. Kelly, Nicholas E. Harrison

**Affiliations:** * Indiana University School of Medicine Emergency Medicine Residency, Indianapolis, Indiana; † Indiana University School of Medicine, Department of Emergency Medicine, Indianapolis, Indiana

**Keywords:** cardiac arrest, case report, myocardial Infarction, terminal QRS distortion

## Abstract

**Introduction:**

Peripartum cardiac arrest is increasing in incidence. While pulmonary embolism (PE) remains an important cause of peripartum morbidity and mortality, other cardiovascular emergencies such as myocardial infarction (MI) are now the leading cause of pregnancy-related death. Emergency physicians (EP) need to be well versed in subtle electrocardiographic (ECG) signs of coronary ischemia to better care for peripartum patients in cardiac arrest.

**Case Report:**

A 38-year-old gravida 2 parity1 female three days post-partum presented in cardiac arrest. After approximately 12 minutes of Advanced Cardiac Life Support including electric defibrillation, the patient experienced sustained return of spontaneous circulation. The physician team was primarily concerned for PE based on an initial ECG demonstrating terminal QRS distortion in V2 but no ST-segment elevation myocardial infarction (STEMI). Computed tomography angiography (CTA) of the chest did not reveal PE. Repeat ECG after CTA demonstrated STEMI criteria, and the patient was emergently taken to the cardiac catheterization laboratory where she was found to have 99% occlusion of the left anterior descending artery.

**Conclusion:**

Emergency physicians should have a high index of suspicion for MI when managing peripartum patients in cardiac arrest. The ECG findings specific for coronary-occlusive acute MI but not included in the classic STEMI criteria increase accuracy and prevent delays in diagnosis; however, the clinical uptake of this paradigm has been slow. Early recognition of terminal QRS distortion can help EPs more rapidly diagnose the etiology of cardiac arrest.

CPC-EM Capsule What do we already know about this clinical entity?

*Peripartum mortality is increasing in incidence, although it remains rare.*

What makes this presentation of disease reportable?

*There are other electrocardiogram patterns apart from ST-elevation myocardial infarction (STEMI) suggestive of occlusive myocardial infarction such as terminal QRS distortion.*

What is the major learning point?

*In the correct clinical setting, terminal QRS distortion is specific for occlusive MI and requires emergent cardiology consultation/intervention.*

How might this improve emergency medicine practice?

*Identifying terminal QRS distortion can help clinicians more expediently identify the cause of cardiac arrest and facilitate appropriate resuscitation.*

## INTRODUCTION

The rate of pregnancy-related mortality has more than doubled in the past 30 years. In 1987, 7.2 deaths per 100,000 live births were pregnancy related; by 2017, pregnancy-related death accounted for 17.3 deaths per 100,000 live births.^1^ Given these epidemiological trends, emergency physicians (EP) are increasingly likely to provide care to peripartum patients in cardiac arrest.

Previously, pulmonary embolism (PE) was the leading cause of maternal death following a live birth.^2^ More recent research, however, indicates that complications of cardiovascular disease (ie, relating to coronary artery disease, hypertension, pulmonary hypertension, congenital valvular disease, and/or vascular malformations) are now the leading cause of pregnancy-related death.^3^ This shift is thought to be driven by increasing maternal age and worsening population cardiovascular health.^4^ We present a case study of peripartum cardiac arrest due to myocardial infarction (MI) and demonstrate the need for EPs to be facile with subtle electrocardiogram (ECG) findings suggestive of acute MI not captured by the traditional ST-elevation myocardial infarction (STEMI) criteria.

## CASE REPORT

A 38-year-old gravida 2 parity1 female with past medical history of hypertension and gestational diabetes presented in cardiac arrest three days postpartum. Labor and delivery course was notable for preeclampsia with severe features. The patient experienced a ventricular fibrillation arrest, was intubated, and underwent 10 minutes of cardiac pulmonary resuscitation with one cardiac defibrillation at which point return of spontaneous circulation (ROSC) was obtained. Four minutes later, the patient experienced a second ventricular fibrillation cardiac arrest, and ROSC was once again obtained with one additional round of Advanced Cardiac Life Support without defibrillation.

**Image 1.**
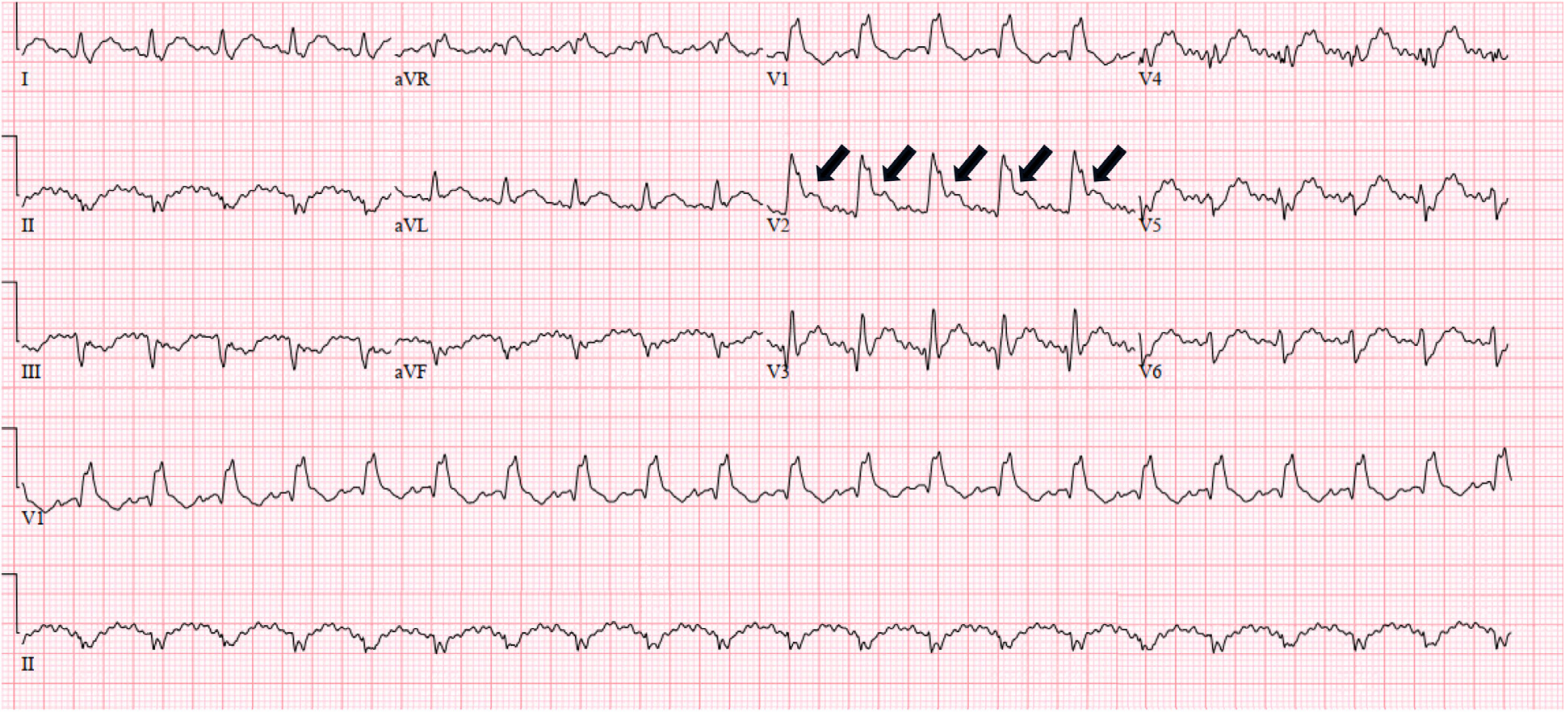
Initial electrocardiogram in a patient post-cardiac arrest demonstrating terminal QRS distortion (arrows) in lead V2 which is not captured by traditional ST-segment elevation myocardial infarction criteria.

Post-arrest, the patient's rhythm was sinus tachycardia with a heart rate of 119 beats per minute and blood pressure of 122/85 millimeters of mercury. Point-of-care ultrasound revealed a dilated right ventricle and globally reduced systolic function. Point-of-care ultrasound did not demonstrate any free fluid within the peritoneum. Initial ECG demonstrated a new incomplete right bundle branch block, terminal QRS distortion in V2, and inferior ST depression (Image 1). The physician team was initially most concerned for massive PE in the postpartum period and decided to proceed with computed tomography angiography (CTA) of the chest.

**Image 2.**
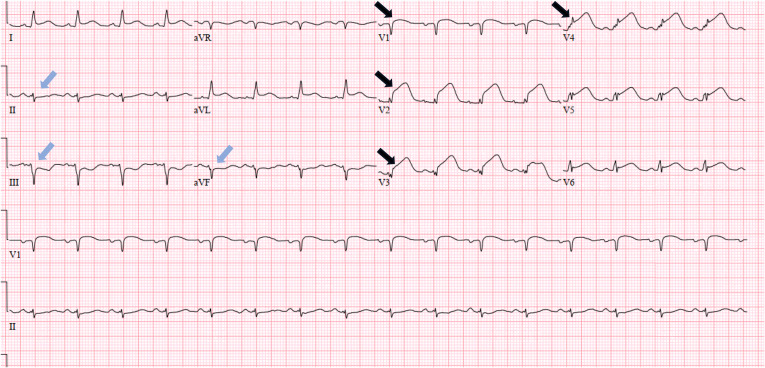
Repeat electrocardiogram in a patient post-cardiac arrest demonstrating ST-segment elevation in leads V1-V4 (black arrows) with reciprocal depressions in the inferior leads (blue arrows) consistent with ST-segment elevation myocardial infarction criteria.

The CTA was negative for PE. Subsequently, serial increase in high-sensitivity troponin (5,480 nanograms per liter (ng/L) (reference range: <3 ng/L–34 ng/L) led to a repeat ECG being obtained, which demonstrated a STEMI pattern (Image 2). The patient was taken to the cardiac catheterization lab where she was found to have a 99% occlusion of the left anterior descending artery with thrombolysis in myocardial infarction (TIMI) 2 flow. Percutaneous coronary intervention was performed with TIMI 3 flow thereafter.

## DISCUSSION

The presented case scenario highlights the evolving epidemiology of peripartum cardiac arrest and need for EPs to be aware of ECG findings consistent with acute MI not included in the traditional STEMI criteria. Insufficient sensitivity of the STEMI criteria to diagnose MI that would benefit from emergent percutaneous coronary intervention (PCI) has led to the development of a new ECG paradigm called occlusive myocardial infarction (OMI).^5^ Occlusive myocardial infarction criteria include the traditional STEMI patterns but go further to include other ECG patterns specific for MIs. These other ECG patterns include Wellens sign, hyperacute T-waves, terminal QRS distortion, among others, and are commonly missed under the STEMI paradigm.^6^

Although traditionally thought of as the most-evidenced ECG pattern of OMI, STEMI criteria are not sufficiently sensitive to reliably diagnose OMI. In fact, multiple studies demonstrate that almost 30% of patients who present with acute coronary syndrome (ACS) but without clear STEMI pattern have acute coronary occlusion.^7^ ^,^ ^8^ The OMI criteria have similar specificity but approximately double the sensitivity of STEMI criteria, allowing EPs to reliably identify more patients that would benefit from emergent PCI. Moreover, use of the OMI criteria reduces time to catheterization for angiographically significant lesions when compared to STEMI criteria alone.^9^

Terminal QRS distortion is very specific for OMI yet remains one of the least discussed ECG patterns associated with ACS. Terminal QRS distortion is defined as the absence of both S and J waves in the anterior precordial leads and is clearly visible in lead V2 of Image 1. Terminal QRS distortion has been found to be 100% specific to left-anterior descending artery occlusion and should never be misinterpreted as benign early repolarization.^10^ Anecdotally, our professional experiences suggest that terminal QRS distortion has received far less discussion and educational emphasis compared to other ECG findings consistent with OMI such as Wellens sign or hyperacute T-waves. Earlier angiography and intervention could have potentially been facilitated by noting the terminal QRS distortion pattern on the initial ECG.

While the STEMI paradigm remains the most common ECG classification relating to acute coronary ischemia, the American College of Cardiology now recognizes multiple ECG patterns as STEMI equivalents.^11^ This recognition of multiple ECG patterns consistent with angiographically significant coronary lesions lends further evidence to the OMI paradigm.

Moving forward, EPs should become more comfortable introducing this novel paradigm into their clinical decision-making when presented with patients at risk of ACS, peripartum or otherwise. Familiarity with terminal QRS distortion and other ECG patterns consistent with OMI will benefit EPs when confronted with peripartum patients who present with undifferentiated chest pain, dyspnea, shock, or cardiac arrest. Given the rising incidence of peripartum cardiovascular disease and associated mortality, EPs should expect to care for more patients experiencing ACS and critical illness in the peripartum period. In these scenarios, identifying terminal QRS distortion (and other ECG patterns of OMI) can provide critical diagnostic information and help expedite PCI or thrombolysis.

## CONCLUSION

Cardiac arrest occurring in the peripartum period is a challenging clinical scenario for the emergency physician. Complications from cardiovascular disease are the leading cause of pregnancy-related death. Consideration of terminal QRS distortion and other non-STEMI ECG findings suggestive of OMI may help emergency physicians more appropriately identify post-cardiac arrest patients who would benefit from emergent PCI.
